# Fixed-dose combination *versus* multi-pill combination antihypertensive therapy in medication-defined apparent treatment-resistant hypertension: real-world evidence on healthcare utilization and health-related quality of life

**DOI:** 10.3389/fphar.2026.1903129

**Published:** 2026-09-11

**Authors:** Eissa A. Jafari

**Affiliations:** Department of Pharmacy Practice, College of Pharmacy, Jazan University, Jazan, Saudi Arabia

**Keywords:** apparent treatment-resistant hypertension, fixed-dose combination antihypertensives, healthcare utilization, health-related quality of life, hypertension, multi-pill combination antihypertensives

## Abstract

**Background:**

Fixed-dose combination (FDC) antihypertensive therapy is recommended by hypertension guidelines to improve adherence and blood pressure control. However, its association with healthcare utilization and health-related quality of life (HRQoL) among patients with apparent treatment-resistant hypertension (aTRH) remains unexplored. This study evaluated the association of FDC *versus* multi-pill combination (MPC) antihypertensive therapy with healthcare utilization and HRQoL among United States (US) adults with medication-defined aTRH**.**

**Methods:**

We conducted a pooled cross-sectional study using data from the Medical Expenditure Panel Survey (2013–2022). Medication-defined aTRH was defined as adults aged ≥18 years with a hypertension diagnosis who were prescribed ≥4 antihypertensive medications from different classes. Medication-defined aTRH patients were then categorized as FDC users if they were prescribed at least one FDC antihypertensive product and as MPC users if they were prescribed antihypertensive classes as separate pills. Healthcare utilization outcomes included office-based visits, outpatient department visits, prescription fill, emergency department visits, home health visits, and inpatient night. HRQoL outcomes included Physical Component Summary (PCS) and Mental Component Summary (MCS) scores. Inverse probability of treatment weighting (IPTW) was performed to balance covariates. IPTW negative binomial and linear regression models were used to assess the associations between FDC antihypertensive therapy *versus* MPC antihypertensive therapy and healthcare utilization and HRQoL, respectively.

**Results:**

The study included 3,131 adults with medication-defined aTRH, of whom 1,518 were FDC users and 1,613 were MPC users. In IPTW models, FDC use was associated with lower rates of office-based visits (rate ratio [RR] = 0.760; 95% confidence interval [CI]: 0.683–0.846), prescription fills (RR = 0.854; 95% CI: 0.791–0.921), emergency department visits (RR = 0.667; 95% CI: 0.551–0.807), and inpatient nights (RR = 0.596; 95% CI: 0.403–0.879). FDC use was also associated with a higher PCS score; IPTW model (β = 2.686; 95% CI: 1.589–3.783). No significant association was observed with MCS score.

**Conclusion:**

Among US adults with medication-defined aTRH, FDC antihypertensive therapy was associated with lower healthcare utilization and better physical HRQoL compared with MPC therapy. These findings suggest that an FDC-containing regimen may represent a valuable treatment-simplification strategy in patients requiring multiple antihypertensive classes.

## 1 Introduction

Hypertension remains one of the leading modifiable risk factors for cardiovascular morbidity and mortality worldwide (Palaniappan et al., 2026; Wu et al., 2025). Despite the increased awareness of hypertension and the availability of effective antihypertensive therapies, blood pressure control remains suboptimal in a large proportion of treated hypertension patients (Muntner et al., 2020; Bakris et al., 2014). Among these are patients with apparent treatment-resistant hypertension (aTRH). aTRH affects about 4%–30% of treated hypertension patients and is defined as requiring ≥4 antihypertensive classes to achieve blood pressure control (Noubiap et al., 2019; Calhoun et al., 2008). aTRH represents a high-risk hypertension phenotype associated with increased risk of adverse cardiovascular events, renal diseases, and all-cause mortality (Smith et al., 2014; Muntner et al., 2014; Irvin et al., 2014). Patients with aTRH often have multiple comorbidities such as diabetes, dyslipidemia, coronary heart disease, chronic kidney disease, and heart failure, which further increase their risk of adverse health outcomes and healthcare utilization, and impair their health-related quality of life (HRQoL) (Jafari et al., 2024; Alshahawey et al., 2024; Bakris et al., 2024; Carris et al., 2016).

One of the major challenges in aTRH management is regimen complexity. Patients with aTRH require multiple antihypertensive classes, exposing them to high pill burden, multiple daily dosing schedules, frequent prescription refills, and cumulative exposure to treatment-related adverse effects, all of which may undermine medication adherence and persistence (Yahr et al., 2023; Durand et al., 2017). Medication nonadherence contributes to poor blood pressure control, greater risk of cardiovascular events, and higher healthcare utilization (Li et al., 2021; Feng et al., 2022). Accordingly, strategies that simplify antihypertensive regimens while maintaining therapeutic efficacy and intensity are needed in this high-risk population. Fixed-dose combination (FDC) antihypertensive products, which combine ≥2 antihypertensive agents in a single pill, represent a strategy that is recommended in hypertension management to improve adherence and blood pressure control (Kawalec et al., 2018; Mobley et al., 2023; Salam et al., 2019). These advantages may be particularly relevant in patients with aTRH, who often require long-term multidrug regimens and may benefit from reducing treatment burden.

Several studies in the general hypertension population have shown that FDC antihypertensive therapy is associated with better adherence, persistence, blood pressure control, and cardiovascular outcomes compared with equivalent multi-pill combination (MPC) regimens (Li et al., 2021; Feng et al., 2022; Kawalec et al., 2018; Mobley et al., 2023; Salam et al., 2019). Additionally, FDC antihypertensive therapy was shown to be associated with lower healthcare utilization and total healthcare costs compared with MPC therapy (Jafari, 2026a; Zhang et al., 2025; Tung et al., 2015; Hess et al., 2008). Despite this growing body of literature, these studies have focused on general hypertensive cohorts, rather than patients with aTRH. Patients with aTRH are likely to have greater healthcare utilization, including more emergency department visits and hospitalizations, and may also experience a substantial prescription burden (Bakris et al., 2024; Flack et al., 2024). Yet, the extent to which FDC *versus* MPC antihypertensive regimens are associated with differences in healthcare utilization in this population remains unexplored.

In addition to health-service outcomes, HRQoL is an important patient-centered outcome in hypertension management. Although hypertension is often viewed as an asymptomatic disease, treatment complexity, medication adverse effects, and awareness of difficult-to-control blood pressure may adversely influence perceived physical and mental wellbeing (Wenger, 1988; Korhonen et al., 2011; Schmieder et al., 2013). FDC antihypertensive therapy has been linked to improved HRQoL in general hypertension populations, with studies reporting improvement in physical and mental health scores and self-rated health after regimen simplification with FDC (Bramlage et al., 2010; Schmieder et al., 2017; Karpov et al., 2015; Karpov and physicians, 2017). However, these findings may not be generalizable to aTRH, a phenotype with greater comorbidity and treatment burden (Jafari et al., 2024; Alshahawey et al., 2024; Carris and Smith, 2015). Evidence on HRQoL in aTRH remains limited. A prior Medical Expenditure Panel Survey (MEPS) study showed that adults with aTRH had worse physical HRQoL than those with non-aTRH, while mental HRQoL was similar between groups (Carris et al., 2016). To date, however, no study has evaluated the association of FDC *versus* MPC antihypertensive therapy with healthcare utilization and HRQoL among adults with aTRH.

To address this gap, this study aimed to evaluate the association of FDC *versus* MPC antihypertensive therapy with healthcare utilization and HRQoL among United States (US) adults with medication-defined aTRH using pooled MEPS data from 2013 to 2022. Findings from this study may provide evidence on the association of FDC antihypertensive therapy with healthcare utilization and HRQoL in adults with medication-defined aTRH.

## 2 Methods

### 2.1 Study design and data source

This was a pooled cross-sectional study using data from the MEPS Household Component from 2013 through 2022. MEPS is a nationally representative survey of the US civilian noninstitutionalized population that collects detailed information on demographic characteristics, health insurance, socioeconomic status, medical conditions, prescription medications, healthcare utilization, expenditures, and HRQoL. MEPS uses a complex multistage probability sampling design, with person-level weights, strata, and primary sampling units provided to generate nationally representative estimates (Jafari, 2026a; Cohen et al., 1996). For this analysis, data were obtained from the Full-Year Consolidated files, Medical Conditions files, and Prescribed Medicines files. Files were linked at the person-year level using the unique participant identifier.

### 2.2 Study population

Participants were included in the study if they were aged ≥18 years, had a hypertension diagnosis, and were prescribed antihypertensive medications during the survey year. Hypertension was identified from the MEPS Medical Conditions files using International Classification of Diseases, Ninth and Tenth Revision diagnosis codes. Individuals with pregnancy-related diagnoses were excluded.

Patients were classified as having medication-defined aTRH if they were prescribed four or more different antihypertensive classes during the same survey year. Because MEPS does not include blood pressure measurements and does not provide sufficiently complete prescription timing and day-supply information to reliably establish overlapping treatment exposure, concurrent use of all four antihypertensive classes could not be confirmed. Therefore, this definition should be interpreted as a medication-defined proxy for aTRH rather than confirmed treatment-resistant hypertension. To reduce risk of misclassification from short-term or incidental medication use and increase the likelihood that identified participants were receiving ongoing multidrug antihypertensive therapy, participants were additionally required to have at least two prescription refills during the survey year. Participants were further required to have complete data on healthcare utilization and HRQoL.

### 2.3 Exposure variable

The exposure variable was antihypertensive regimen type, classified as FDC therapy *versus* MPC therapy. Both exposure groups were required to be prescribed four or more different antihypertensive classes. FDC therapy was defined as the prescribing of at least one antihypertensive product containing two or more active antihypertensive agents in a single pill. On the other hand, MPC therapy was defined as the prescribing of antihypertensive classes as separate single-pill products without an FDC product.

### 2.4 Study outcomes

Healthcare utilization outcomes were measured as annual counts during the same MEPS survey year. Outcomes included office-based visits (OBTOTV), outpatient department visits (OPTOTV), prescription fills (RXTOT), emergency department visits (ERTOT), home health visits (HHTOTD), and inpatient night (IPNGTD). HRQoL was assessed using Physical Component Summary and Mental Component Summary scores derived from the Veterans RAND-12 health survey. PCS (VPCS) and MCS (VMCS) were analyzed as continuous outcomes, with higher scores indicating better HRQoL (Selim et al., 2022; Cheak et al., 2009).

### 2.5 Covariates

Covariates included age category, sex, race/ethnicity, education, insurance coverage, poverty category, marital status, geographic region, physical activity, smoking, dyslipidemia, chronic kidney disease, diabetes, stroke, myocardial infarction, heart failure, coronary heart disease, chronic obstructive pulmonary disease, asthma, Alzheimer’s disease and related dementia, osteoarthritis, anxiety, and depression.

### 2.6 Statistical analysis

Descriptive statistics were used to compare patient characteristics between FDC and MPC users before and after the inverse probability of treatment weighting (IPTW) approach. Categorical variables were reported as frequencies and weighted percentages, while continuous variables were reported as weighted means with standard deviations. Between-group balance was assessed using standardized mean differences (SMDs), with values below 0.10 indicating acceptable balance.

Propensity scores estimating the probability of receiving FDC antihypertensive therapy were generated using a multivariable-adjusted logistic regression model including age, sex, race/ethnicity, education, insurance coverage, geographic region, physical activity, smoking, survey year, dyslipidemia, chronic kidney disease, diabetes, stroke, myocardial infarction, heart failure, and coronary heart disease. Stabilized IPTWs were derived from the propensity scores. To reduce the influence of extreme weights, upper-tail truncation was applied by replacing weights above the 95th percentile with the 95th-percentile value. Final analysis weights for IPTW analyses were generated by multiplying the truncated stabilized IPTW weights by the MEPS person-level survey weights. Covariate balance after IPTW was reassessed using SMDs. Propensity-score overlap was examined graphically (Supplementary Figure S1), and weight distributions were evaluated as additional weighting diagnostics (Supplementary Table S1).

Unadjusted comparisons of healthcare utilization and HRQoL outcomes between FDC and MPC users were performed using survey-weighted negative binomial regression models and survey-weighted linear regression models, respectively.

The association of FDC antihypertensive therapy with healthcare utilization outcomes was analyzed using survey-weighted negative binomial regression models. Results were reported as rate ratios (RR) with 95% confidence intervals (CIs) and p-values. The association of FDC antihypertensive therapy with HRQoL outcomes was analyzed using survey-weighted linear regression models and reported as beta coefficients (β), with standard errors (SE), 95% CI, and p-values. Two analyses were used: multivariable-adjusted regression analysis using the original cohort and IPTW regression analysis using the balanced pseudo-population. All analyses accounted for the MEPS complex survey design, including person-level weights, strata, and primary sampling units. Statistical analyses were performed using SAS version 9.4 (SAS Institute, Cary, NC) and R version 4.3.3 (R Foundation for Statistical Computing, Vienna, Austria).

## 3 Results

### 3.1 Data preparation and cohort selection

**FIGURE 1:**
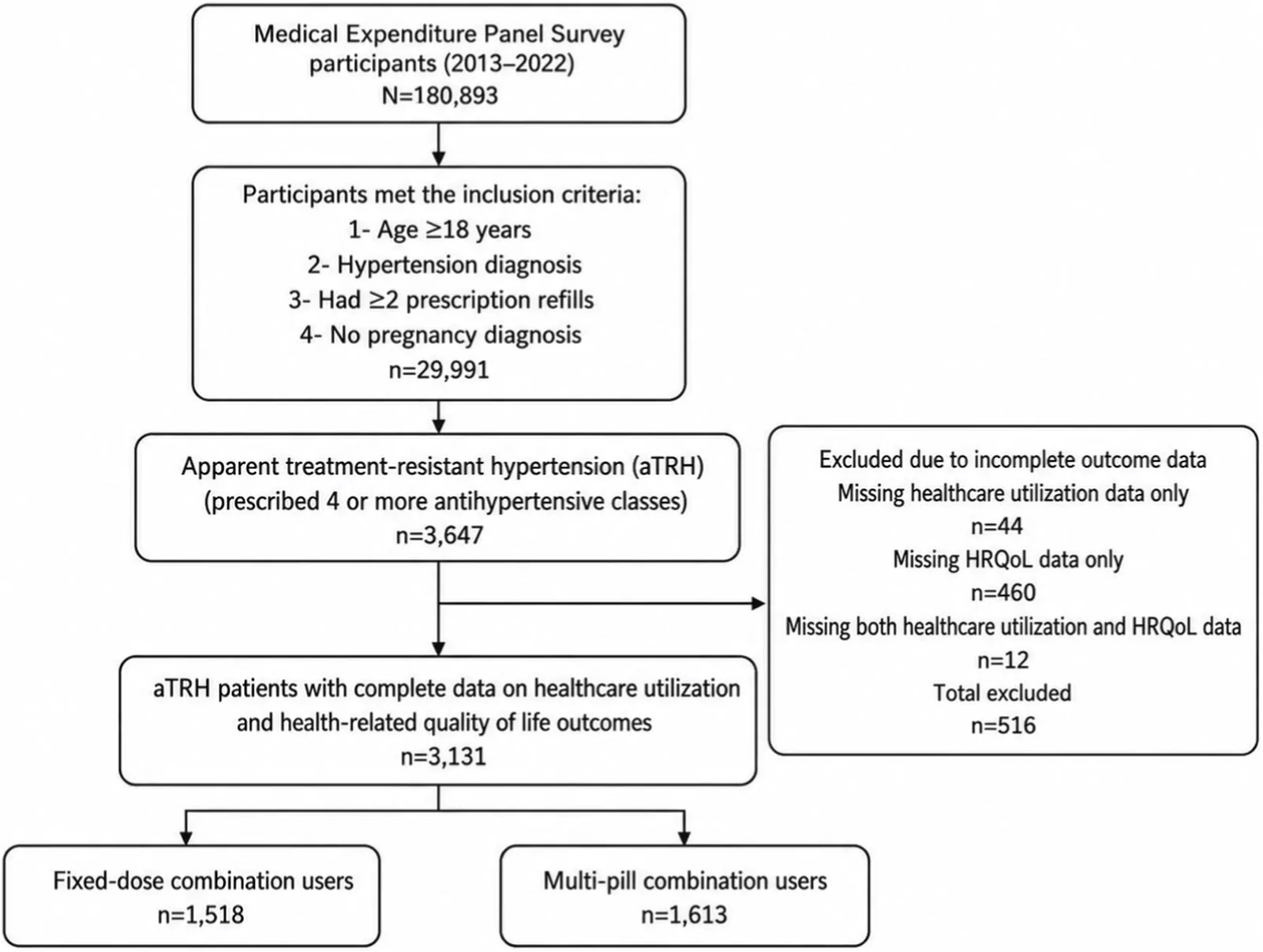
Flowchart of data preparation and cohort selection.

Figure 1 shows the cohort selection process. From 180,893 MEPS participants between 2013 and 2022, 29,991 adults met the initial inclusion criteria: age ≥18 years, hypertension diagnosis, ≥2 prescription refills, and no pregnancy diagnosis. Among these eligible adults with hypertension, 3,647 met the criteria for medication-defined aTRH. Of the 516 participants excluded because of missing outcome data, 44 had only missing healthcare utilization data, 460 had only missing HRQoL data, and 12 had missing data for both outcomes. The final analytic sample included 3,131 adults with medication-defined aTRH. Of these, 1,518 were FDC users and 1,613 MPC users.

### 3.2 Patient characteristics

Table 1 summarizes the patient characteristics for the overall cohort and by FDC and MPC users before and after IPTW. The mean age of the cohort was 68 years, and most participants were aged ≥65 years (64%). The female sex was 55%, and the largest racial/ethnic groups were non-Hispanic White (50%), followed by non-Hispanic Black (31%) and Hispanic participants (13%). Common comorbidities included dyslipidemia (78%), diabetes (46%), osteoarthritis (36%), coronary heart disease (28%), stroke (19%), and myocardial infarction (18%).

**TABLE 1:** Patient characteristics.

Characteristics	Total population	Before IPTW			After IPTW		
	Overall N = 3,131 (wt%)	MPC n = 1,613 (wt%)	FDC n = 1,518 (wt%)	SMD	MPC n = 1,613 (wt%)	FDC n = 1,518 (wt%)	SMD
Age (SD)	68 (12)	70 (12)	66 (12)	0.294	68 (12)	67 (11)	0.083
Age category							
18–39	61 (2)	24 (2)	37 (2)	0.068	32 (2)	29 (2)	0.007
40–64	1,054 (34)	464 (29)	590 (39)	0.215	533 (33)	508 (33)	0.009
≥65	2,016 (64)	1,125 (69)	891 (59)	0.232	1,048 (65)	981 (65)	0.007
Sex							
Male	1,396 (45)	766 (47)	630 (42)	0.121	724 (45)	673 (44)	0.011
Female	1,735 (55)	847 (53)	888 (58)	0.121	889 (55)	845 (56)	0.011
Race/Ethnicity							
Hispanic	397 (13)	214 (13)	183 (12)	0.036	205 (13)	187 (12)	0.012
Non-Hispanic White	1,574 (50)	821 (51)	753 (50)	0.026	809(50)	740 (49)	0.027
Non-Hispanic Black	974 (31)	485 (30)	489 (32)	0.046	503 (31)	504 (33)	0.042
Non-Hispanic Asian	109 (3)	47 (3)	62 (4)	0.064	55 (3)	49 (3)	0.010
Other races	77 (3)	46 (3)	31 (2)	0.052	41 (3)	38 (3)	0.003
Education							
No degree	647 (21)	346 (22)	301 (20)	0.040	331 (21)	310 (20)	0.004
High school diploma	1,517 (48)	797 (48)	720 (47)	0.040	782 (48)	743 (49)	0.010
Some college/associate degree	379 (12)	188 (12)	191 (13)	0.028	195 (12)	186 (12)	0.007
Bachelor’s degree/higher education	588 (19)	282 (18)	306 (20)	0.068	305 (19)	279 (19)	0.014
Insurance coverage							
Private	1,474 (47)	671 (41)	803 (53)	0.228	756 (47)	702 (47)	0.012
Public	1,578 (50)	915 (57)	663 (44)	0.263	818 (51)	779 (51)	0.013
Uninsured	79 (3)	27 (2)	52 (3)	0.111	39 (2)	37 (2)	0.002
Poverty category							
Poor	645 (21)	358 (22)	287 (19)	0.081	339 (21)	302 (20)	0.027
Near poor	257 (8)	145 (9)	112 (8)	0.059	134 (8)	125 (8)	0.002
Low income	503 (16)	287 (18)	216 (14)	0.097	279 (17)	229 (15)	0.060
Middle income	845 (27)	402 (25)	443 (29)	0.096	404 (25)	439 (29)	0.087
High income	881 (28)	421 (26)	460 (30)	0.093	457 (28)	423 (28)	0.011
Marital status							
Married	1,465 (47)	709 (44)	756 (50)	0.117	730 (45)	727 (48)	0.053
Divorced/widowed/separated	1,332 (42)	721 (45)	611 (40)	0.090	693 (43)	652 (43)	0.000
Never married	334 (11)	183 (11)	151 (10)	0.045	190 (12)	139 (9)	0.087
Region							
Northwest	494 (16)	255 (16)	239 (16)	0.002	252 (15)	239 (15)	0.003
Midwest	674 (21)	381 (24)	293 (19)	0.105	353 (22)	333 (22)	0.000
South	1,462 (47)	701 (43)	761 (50)	0.134	753 (47)	708 (47)	0.001
West	501 (16)	276 (17)	225 (15)	0.063	255 (16)	238 (16)	0.001
Physical exercise	1,099 (35)	537 (33)	562 (37)	0.078	572 (35)	537 (35)	0.002
Smoking status	2,130 (68)	1,055 (65)	1,075 (71)	0.116	1,098 (68)	1,036 (68)	0.004
Dyslipidemia	2,455 (78)	1,322 (82)	1,133 (75)	0.178	1,265 (78)	1,184 (78)	0.009
Chronic kidney disease	157 (5)	128 (8)	29 (2)	0.281	82 (5)	93 (6)	0.046
Diabetes	1,448 (46)	825 (51)	623 (41)	0.204	749 (47)	710 (47)	0.007
Stroke	604 (19)	401 (25)	203 (13)	0.295	315 (20)	301 (20)	0.007
Myocardial infarction	551 (18)	385 (24)	166 (11)	0.346	287 (18)	291 (19)	0.035
Heart failure	246 (8)	193 (12)	53 (4)	0.321	128 (8)	134 (9)	0.033
Coronary heart disease	868 (28)	602 (37)	266 (18)	0.455	448 (28)	425 (28)	0.005
COPD	508 (16)	298 (19)	210 (14)	0.126	269 (17)	235 (16)	0.033
Asthma	547 (18)	288 (18)	259 (17)	0.021	286 (18)	283 (19)	0.024
ADRD	541 (17)	337 (21)	204 (13)	0.199	295 (18)	244 (16)	0.059
Osteoarthritis	1,140 (36)	593 (37)	547 (36)	0.015	581 (36)	562 (37)	0.021
Anxiety	529 (17)	264 (16)	265 (18)	0.029	263 (16)	268 (18)	0.035
Depression	361 (12)	206 (13)	155 (10)	0.080	193 (12)	165 (11)	0.035

Abbreviation: FDC, Fixed-dose combination; MPC, Multi-pill combination; IPTW, inverse probability of treatment weighting; SMD, Standardized mean difference; ADRD, Alzheimer’s disease and related dementia; GERD, Gastroesophageal reflux disease; COPD, Chronic obstructive pulmonary disease; wt%, Weighted percentage; SD, standard deviation.

**FIGURE 2:**
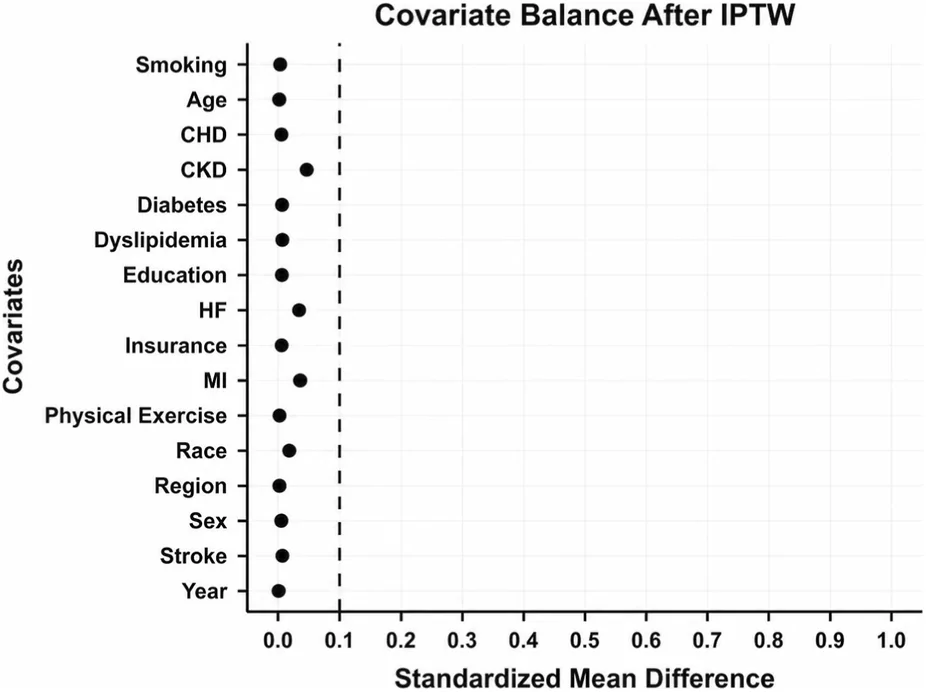
Covariates balance after IPTW. Abbreviations: IPTW, inverse probability of treatment weighting; CHD, coronary heart disease; CKD, chronic kidney disease; HF, heart failure; MI, myocardial infarction.

Before IPTW, several covariates were imbalanced between FDC and MPC users. However, after applying IPTW, all covariates were balanced between the FDC and MPC groups, with SMD below 0.10, indicating acceptable covariate balance (Table 1; Figure 2). The distribution of antihypertensive drug classes and the number of antihypertensive classes by treatment group are presented in Supplementary Table S2; Supplementary Table S3, respectively.

### 3.3 FDC and healthcare utilization outcomes

Table 2 presents the survey-weighted unadjusted comparison of healthcare utilization outcomes between FDC and MPC users. FDC users had significantly lower rate of office-based visits (11.95 vs. 17.67; *p* < 0.0001), outpatient department visits (1.65 vs. 2.79; *p* = 0.0005), prescription fills (40.72 vs. 53.76; *p* < 0.0001), emergency department visits (0.31 vs. 0.61; *p* < 0.0001), home health visits (6.16 vs. 13.54; *p* < 0.0001), and inpatient nights (1.21 vs. 2.84; *p* < 0.0001) compared with MPC users.

**TABLE 2:** Weighted comparison of healthcare utilization outcomes by antihypertensive regimen.

Outcome	MPC Mean (SE)	FDC Mean (SE)	*p*-value
Office-based visits	17.67 (0.61)	11.95 (0.45)	<0.0001
Outpatient department visits	2.79 (0.24)	1.65 (0.20)	0.0005
Prescription fills	53.76 (1.16)	40.72 (1.12)	<0.0001
Emergency room visits	0.61 (0.04)	0.31 (0.02)	<0.0001
Home health visits	13.54 (1.32)	6.16 (1.00)	<0.0001
Inpatient nights	2.84 (0.26)	1.21 (0.21)	<0.0001

Abbreviations: FDC, fixed-dose combination; MPC, multi-pill combination; SE, standard error.

In the IPTW analysis, FDC users had significantly lower rates of office-based visits (RR = 0.760; 95% CI: 0.683–0.846; *p* < 0.0001), prescription fills (RR = 0.854; 95% CI: 0.791–0.921; *p* = 0.0001), emergency department visits (RR = 0.667; 95% CI: 0.551–0.807; *p* < 0.0001), and inpatient nights (RR = 0.596; 95% CI: 0.403–0.879; *p* = 0.0093) compared with MPC users. No statistically significant association was observed for outpatient department visits (RR = 0.816; 95% CI: 0.577–1.154; *p* = 0.2498) or home health visits (RR = 0.729; 95% CI: 0.510–1.041; *p* = 0.0821) (Table 3). Similar findings were observed in multivariable-adjusted models, except for home health visits.

**TABLE 3:** Results of adjusted multivariate and IPTW models evaluating the association of FDC *versus* MPC with healthcare utilization.

Outcome	Adjusted multivariate model			IPTW model		
​	RR	95% CI	*p*-value	RR	95% CI	*p*-value
Office-based visits	0.777	(0.702–0.860)	<0.0001	0.760	(0.683–0.846)	<0.0001
Outpatient department visits	0.815	(0.585–1.135)	0.2245	0.816	(0.577–1.154)	0.2498
Prescription fills	0.839	(0.787–0.895)	<0.0001	0.854	(0.791–0.921)	0.0001
Emergency department visits	0.655	(0.548–0.784)	<0.0001	0.667	(0.551–0.807)	<0.0001
Home health visits	0.656	(0.461–0.932)	0.0188	0.729	(0.510–1.041)	0.0821
Inpatient nights	0.641	(0.443–0.926)	0.0179	0.596	(0.403–0.879)	0.0093

Abbreviations: FDC, fixed-dose combination; MPC, multi-pill combination; IPTW, inverse probability of treatment weighting; RR, rate ratio; CI: confidence interval.

### 3.4 FDC and health-related quality of life outcomes

For HRQoL outcomes, FDC users had a significantly higher unadjusted PCS score (41.69 vs. 36.05; *p* < 0.0001) and MCS score (51.60 vs. 50.30; *p* = 0.0049) compared with MPC users (Table 4).

**TABLE 4:** Weighted comparison of health-related quality-of-life outcomes by antihypertensive regimen.

Outcome	MPC Mean (SE)	FDC Mean (SE)	*p*-value
PCS	36.05 (0.36)	41.69 (0.39)	<0.0001
MCS	50.30 (0.33)	51.60 (0.35)	0.0049

Abbreviations: FDC, fixed-dose combination; MPC, multi-pill combination; SE, standard error; PCS, physical component summary; MCS, mental component summary.

In the IPTW analysis, FDC use remained significantly associated with a higher PCS score (β = 2.686; SE = 0.558; 95% CI: 1.589–3.783; *p* < 0.0001) compared with MPC use. However, no statistically significant association was observed for MCS score after IPTW adjustment (β = 0.068; SE = 0.526; 95% CI: −0.965–1.101; *p* = 0.8971) (Table 5). Similar findings were observed in the multivariable-adjusted models (Table 5).

**TABLE 5:** Results of adjusted multivariate and IPTW models evaluating the association of FDC *versus* MPC with health-related quality of life.

Outcome	Adjusted multivariate model				IPTW model			
​	β	SE	95% CI	*p*-value	β	SE	95% CI	*p*-value
PCS	2.762	0.508	(1.763–3.761)	<0.0001	2.686	0.558	(1.589–3.783)	<0.0001
MCS	0.227	0.438	(-0.634–1.089)	0.6044	0.068	0.526	(-0.965–1.101)	0.8971

Abbreviation: FDC, Fixed-dose combination; MPC, Multi-pill combination; IPTW, inverse probability of treatment weighting; β, Beta coefficient; SE, standard error; CI, confidence interval; PCS, Physical component summary; MCS, Mental component summary.

## 4 Discussion

In this nationally representative study of US adults with medication-defined aTRH, FDC antihypertensive therapy was associated with lower healthcare utilization across several healthcare settings, including office-based visits, prescription fills, emergency department visits, and inpatient nights, compared with MPC therapy. In addition, FDC use was also associated with better physical HRQoL, whereas no significant association was observed for mental HRQoL. These findings suggest that among adults with medication-defined aTRH, FDC-containing regimens may be associated with lower care burden and better perceived physical wellbeing.

The lower number of prescription fills observed among FDC users is consistent with prior evidence from general hypertension populations showing fewer fill events with FDC antihypertensive therapy compared with MPC therapy (Jafari, 2026a). This finding may reflect regimen consolidation rather than lower antihypertensive treatment intensity. Since both treatment groups met the medication-defined aTRH criterion of receiving at least four antihypertensive classes, the lower prescription fill burden among FDC users is unlikely to reflect less intensive antihypertensive therapy. Instead, FDC antihypertensive therapy may allow multidrug coverage to be delivered through fewer individual prescriptions, thereby reducing prescription fill burden. This may be particularly meaningful in aTRH, where patients require complex and often long-term multidrug regimens.

The observed lower rates in emergency department visits and inpatient nights among FDC users are clinically plausible and consistent with the established benefits of FDC antihypertensive therapy in hypertension management (Jafari, 2026a; Zhang et al., 2025). Previous studies have associated FDC therapy with better adherence, persistence, and blood pressure control, thereby reducing acute decompensations such as stroke and myocardial infarction that require emergent or inpatient care (Li et al., 2021; Feng et al., 2022; Kawalec et al., 2018; Mobley et al., 2023; Salam et al., 2019). In patients with aTRH, these mechanisms may be relevant as these individuals face regimen complexity, high comorbidity burden, and a greater likelihood of adverse outcomes related to difficult-to-control blood pressure (Smith et al., 2014; Muntner et al., 2014; Irvin et al., 2014; Jafari et al., 2024; Alshahawey et al., 2024; Carris and Smith, 2015). Although MEPS captures all-cause rather than specific cause of healthcare utilization, these reductions cannot be attributed specifically to cardiovascular or hypertension-related events; however, they may still reflect a broader association between FDC use and lower acute healthcare burden among adults with medication-defined aTRH.

The lower rates of office-based visits among FDC users may suggest reduced routine ambulatory care burden in medication-defined aTRH. In MEPS, office-based visits capture encounters in provider clinics, where hypertension follow-up, medication review, treatment titration, and chronic disease management commonly occur. These settings may be affected by antihypertensive regimen complexity, as patients treated with multiple separate agents may require more visits for medication reconciliation, adverse-effect assessment, or follow-up after regimen adjustment. In contrast, FDC-containing regimens may provide similar multidrug coverage with fewer medications, potentially reducing some routine medication-management encounters. This finding is consistent with a prior MEPS analysis in the general hypertension population, where FDC use was also associated with lower office-based visits, compared with MPC therapy (Jafari, 2026a). Although fewer office-based visits may not inherently be desirable, the concurrent reductions in prescription fills, emergency department visits, and inpatient nights among FDC users suggest that this pattern may reflect lower care burden and more efficient hypertension management rather than disengagement from care; however, the cross-sectional design precludes determining the direction of these associations.

In contrast to the office-based findings, outpatient department visits were not significantly different between FDC and MPC users. This pattern may suggest differences in healthcare settings. In MEPS, outpatient department visits generally reflect hospital-based outpatient care rather than routine office-based management and may be driven more by specialty services, diagnostic testing, procedures, or comorbidities-related care than by antihypertensive regimen complexity. Therefore, FDC-related regimen simplification may be more likely to influence office-based utilization than hospital outpatient department utilization. From a health-services perspective, the absence of a significant reduction in outpatient department visits may be favorable, because a decline across all ambulatory settings may suggest underuse of necessary follow-up rather than improved disease management. The preserved outpatient department visit rates, together with lower office-based visits, emergency department visits, and inpatient nights, may suggest a lower routine and acute care burden without broad disengagement from healthcare. This result is also consistent with a prior MEPS analysis in the general hypertension population, in which outpatient department visits were likewise not significantly reduced among FDC users despite lower acute-care utilization (Jafari, 2026a).

Similarly, home health visits were not significantly different between FDC and MPC users, suggesting that this domain of healthcare use may be less influenced by antihypertensive regimen type. In MEPS, home health is captured as a separate category of care delivered at home and often reflects supportive, skilled, or assistance-based services rather than standard office-based management of hypertension. In an older, highly comorbid population with aTRH, home health utilization may be driven more by frailty, disability, cognitive impairment, post-acute care needs, and dependence in daily functioning than by differences in antihypertensive regimen structure. Therefore, the lack of a significant association for home health use may be clinically plausible. This null finding may support the overall results by showing that the association between FDC antihypertensive therapy and lower healthcare utilization was not uniform across all healthcare settings but was apparent in outcomes more plausibly linked to medication-management burden and acute clinical care.

The observed differences in healthcare utilization may also reflect clinical and prescribing factors. Treatment selection in hypertension is influenced not only by blood pressure response but also by comorbidity burden, prior treatment history, clinician prescribing preferences, and formulary access (Leader et al., 2001; Bramlage et al., 2018; O'Hagan et al., 2024). Clinicians may preferentially prescribe MPC regimens to patients requiring frequent dose adjustment, individualized drug selection, or management of more complex cardiovascular conditions, whereas FDC products may be prescribed for patients with more stable hypertension (Bramlage et al., 2018). In addition, formulary access may influence whether an FDC product is available or covered, independently of the patient’s clinical characteristics (O'Hagan et al., 2024). Because MEPS does not include blood pressure measurements, detailed treatment history, or the clinical rationale underlying regimen selection, these factors may partly explain the lower healthcare utilization observed among FDC users and should be considered when interpreting the findings.

The higher PCS score observed among FDC users suggests that regimen simplification may be associated with better physical health status in adults with medication-defined aTRH. This result is consistent with prior studies in general and uncontrolled hypertension populations that linked FDC use to improved patient-reported physical health (Bramlage et al., 2010; Schmieder et al., 2017; Karpov et al., 2015; Karpov and physicians, 2017). Because patients with aTRH require multiple antihypertensive classes, they often face substantial regimen complexity, refill demands, treatment fatigue, and medication-management burden, all of which may adversely affect physical wellbeing (Carris and Smith, 2015). FDC-containing regimens offer a potential means of simplifying medication scheduling and refill coordination. Prior studies have associated lower treatment complexity with better treatment implementation, persistence, and adherence, which could theoretically contribute to more stable blood pressure control and better perceived physical functioning. Moreover, the lower rates of emergency department visits and inpatient nights observed among FDC users may also support better perceived physical functioning by reducing acute health disruptions and the physical burden of urgent or inpatient care. However, these mechanisms cannot be confirmed in MEPS, and reverse causation remains possible, as patients with poorer physical health or greater clinical complexity may have been more likely to receive MPC therapy.

In contrast, FDC use was not associated with a significantly higher MCS score. This pattern is not unexpected, as mental HRQoL in hypertension is likely shaped by a broader set of factors, including depression, anxiety, social support, financial stress, caregiving burden, and psychosocial stressors, many of which may not be altered by whether antihypertensive therapy is prescribed as FDC or MPC. The lack of a significant association with MCS is also consistent with prior MEPS findings showing that adults with aTRH had poorer physical HRQoL but similar mental HRQoL compared with those with no aTRH, suggesting that, within aTRH, regimen-related differences may be more detectable in physical than in mental HRQoL (Carris et al., 2016). Although some studies in general and uncontrolled hypertension populations have reported improvement in emotional burden or mental health score with FDC antihypertensive therapy, those studies were conducted largely in general hypertension populations and may not be generalizable to the aTRH population (Bramlage et al., 2010; Schmieder et al., 2017).

The present study represents a focused extension of our previous MEPS analysis comparing FDC and MPC antihypertensive therapy in the broader population of adults with hypertension receiving multidrug treatment (Jafari, 2026a). While the previous analysis included adults receiving at least two antihypertensive classes, the current study focused on adults receiving four or more classes and meeting a medication-defined aTRH. This distinction is clinically relevant because patients receiving four or more antihypertensive classes face greater regimen complexity and prescription burden, providing a rationale for evaluating FDC-based regimen simplification in this population. The present study also extends the previous analysis by evaluating the association of FDC antihypertensive therapy with physical and mental HRQoL outcomes.

The present findings have important clinical and policy implications. Patients with aTRH require intensive multidrug antihypertensive therapy and are vulnerable to high pill burden and treatment fatigue. An FDC-containing regimen offers an established approach to regimen simplification when clinically appropriate for these patients. In the present study, FDC use was associated with lower utilization across several healthcare settings and higher physical HRQoL; however, because temporality and causality cannot be established, these findings should not be interpreted as evidence that FDC therapy itself reduces healthcare utilization or improves HRQoL. Rather, they provide hypothesis-generating evidence supporting further longitudinal evaluation of FDC-based regimen simplification in aTRH patients. From a policy perspective, this is especially relevant given prior evidence that FDC antihypertensive use has declined in the US over the past decade, including after the 2017 ACC/AHA hypertension guideline update, despite guideline support for regimen simplification (Jafari, 2026b). This gap between guideline recommendations and prescribing practice highlights the need to identify barriers to FDC use and improve access to clinically appropriate FDC products for high-pill-burden patients.

Several limitations should be considered when interpreting these findings. First, because MEPS lacks blood pressure data, this study identified medication-defined aTRH rather than true treatment-resistant hypertension; accordingly, patients with uncontrolled blood pressure while being prescribed three antihypertensive classes were not identified by the study definition. MEPS does not provide prescription timing information to determine overlapping treatment exposure or distinguish treatment switching, which may have resulted in misclassification of medication-defined aTRH. Second, MEPS does not provide a measure of medication adherence; thus, prescription refills do not guarantee that medications were taken as prescribed. Third, treatment regimen classification and study outcomes were measured within the same survey year; therefore, temporality cannot be established, and the findings should therefore be interpreted as an association rather than a causal effect. Although both treatment groups received at least four antihypertensive classes, differences in regimen composition may have influenced treatment selection and outcomes. Fourth, although IPTW and multivariable-adjusted models were used to account for confounders, residual confounding remains possible from unobserved factors such as hypertension severity, disease duration, baseline blood pressure, prior healthcare utilization, prescriber preference, and formulary access. Lastly, the prescription-fill outcome in MEPS reflects total medication use rather than antihypertensive medications specifically, so the lower number of fills observed among FDC users cannot be attributed exclusively to antihypertensive therapy. Similarly, the healthcare utilization outcomes captured in MEPS are all-cause rather than hypertension-specific or cardiovascular-specific, which limits interpretation of the exact clinical reasons underlying differences in emergency department visits and inpatient nights. Despite these limitations, this study used nationally representative data from MEPS, enhancing the generalizability of the findings. To our knowledge, this is the first study evaluating the association of FDC antihypertensive therapy with both healthcare utilization and HRQoL in a hypertension population with medication-defined aTRH.

## 5 Conclusion

In this nationally representative study of US adults with medication-defined aTRH, FDC antihypertensive therapy was associated with lower healthcare utilization and better physical HRQoL compared with MPC therapy. These findings suggest that, among patients requiring multidrug antihypertensive treatment, an FDC-containing regimen may represent a practical treatment-simplification strategy associated with lower care burden and better perceived physical wellbeing. Future longitudinal studies incorporating blood pressure measurements, medication adherence, and clinical cardiovascular outcomes are needed to confirm whether FDC antihypertensive therapy can reduce healthcare burden and improve patient-centered outcomes in aTRH.

## Data Availability

The data used in this study are publicly available from the Medical Expenditure Panel Survey, sponsored by the Agency for Healthcare Research and Quality, and can be accessed at: Medical Expenditure Panel Survey Home. All results relevant to this study are included in the article and are available upon reasonable requests to the corresponding author. Publicly available datasets were analyzed in this study. This data can be found here: https://meps.ahrq.gov/mepsweb/data_stats/download_data_files.jsp.
