# Supplementary material for: Fixed-dose combination versus multi-pill combination antihypertensive therapy in medication-defined apparent treatment-resistant hypertension: real-world evidence on healthcare utilization and health-related quality of life
Source: Front Pharmacol. 2026 Sep 11;17:1903129. doi: 10.3389/fphar.2026.1903129 (PMC13612530; doi:10.3389/fphar.2026.1903129)
Supplement: Supplementary file 1 [file DataSheet1.docx]

**Supplementary Materials**

**Supplementary Figure S1. Propensity score distribution by treatment group before inverse probability of treatment weighting**

**Supplementary Table S1. Inverse probability of treatment weighting diagnostics**

| Total sample size | 3,131 |
| --- | --- |
| Truncation threshold | 95th percentile |
| Participants affected by truncation, n (%) | 156 (4.98%) |
| Maximum stabilized weight before truncation | 8.89 |
| Maximum stabilized weight after truncation | 1.76 |
| Effective sample size after weighting | 2,477 |

**Supplementary Table S2. Distribution of antihypertensive drug classes in treatment groups**

| Drug class | MPC (1,613) | FDC (1,518) |
| --- | --- | --- |
| Beta-blocker | 1,384 (86%) | 1,169 (77%) |
| Angiotensin converting enzyme inhibitors | 1,029 (64%) | 1,094 (72%) |
| Calcium channel blocker | 1,189 (74%) | 1,137 (75%) |
| Diuretics | 1,316 (82%) | 1,446 (95%) |
| Angiotensin receptor blocker | 972 (60%) | 1,051 (69%) |
| Centrally acting alpha agonist | 275 (17%) | 213 (14%) |
| Aldosterone receptor antagonist | 356 (22%) | 290 (19%) |
| alpha-1 peripherally acting antagonist | 270 (17%) | 183 (12%) |
| Vasodilators | 369 (23%) | 247 (16%) |
| Miscellaneous antihypertensive | 0 | 310 (20%) |

Abbreviation: MPC: multi-pill combination, FDC: fixed-dose combination

**Supplementary Table S3. Distribution of number of antihypertensive drug classes in treatment groups**

| Number of drug class | MPC (1,613) | FDC (1,518) |
| --- | --- | --- |
| 4 | 1,119 (69%) | 875 (58%) |
| 5 | 351 (22%) | 390 (26%) |
| ≥6 | 143 (9%) | 253 (17%) |

Abbreviation: MPC: multi-pill combination, FDC: fixed-dose combination
